# AIF1L regulates actomyosin contractility and filopodial extensions in human podocytes

**DOI:** 10.1371/journal.pone.0200487

**Published:** 2018-07-12

**Authors:** Mako Yasuda-Yamahara, Manuel Rogg, Kosuke Yamahara, Jasmin I. Maier, Tobias B. Huber, Christoph Schell

**Affiliations:** 1 Department of Medicine IV, Medical Center – University of Freiburg, Faculty of Medicine, University of Freiburg, Freiburg, Germany; 2 Department of Medicine, Shiga University of Medical Science, Otsu, Shiga, Japan; 3 Institute for Surgical Pathology, University Medical Center Freiburg, Freiburg, Germany; 4 BIOSS Center for Biological Signalling Studies, Albert-Ludwigs-University Freiburg, Freiburg, Germany; 5 Department of Medicine III, University Medical Center Hamburg-Eppendorf, Hamburg, Germany; 6 Berta-Ottenstein Programme, Faculty of Medicine, University of Freiburg, Freiburg, Germany; University of Houston, UNITED STATES

## Abstract

Podocytes are highly-specialized epithelial cells essentially required for the generation and the maintenance of the kidney filtration barrier. This elementary function is directly based on an elaborated cytoskeletal apparatus establishing a complex network of primary and secondary processes. Here, we identify the actin-bundling protein allograft-inflammatory-inhibitor 1 like (AIF1L) as a selectively expressed podocyte protein *in vivo*. We describe the distinct subcellular localization of AIF1L to actin stress fibers, focal adhesion complexes and the nuclear compartment of podocytes *in vitro*. Genetic deletion of *AIF1L* in immortalized human podocytes resulted in an increased formation of filopodial extensions and decreased actomyosin contractility. By the use of SILAC based quantitative proteomics analysis we describe the podocyte specific AIF1L interactome and identify several components of the actomyosin machinery such as MYL9 and UNC45A as potential AIF1L interaction partners. Together, these findings indicate an involvement of AIF1L in the stabilization of podocyte morphology by titrating actomyosin contractility and membrane dynamics.

## Introduction

Podocytes are specialized epithelial cells essentially involved in the generation and maintenance of the glomerular filtration barrier [1,2]. These cells show a highly arborized and branched cellular morphology, where primary processes extend from the cell body and elaborate in thin protrusions termed as secondary or foot processes (FPs—[3,4]). FPs of adjacent podocytes interdigitate, generating a dense FP network on the outer surface of glomerular capillaries [4]. Insights from genetic studies and experimental models support the notion that podocytes essentially rely on a diverse and elaborate actin cytoskeleton to maintain their complex morphology and integrity of the kidney filtration barrier [5,6]. For example, mutations in the actin bundling protein *alpha-Actinin-4* lead to progressive podocyte damage finally resulting in a condition termed as focal segmental glomerulosclerosis (FSGS—[7]). Given the prominent localization on the outer surface of glomerular capillaries podocytes essentially rely on a fine controlled adhesion system via specialized complexes commonly termed as focal adhesion [2]. We and others have shown that components of this complex not only fulfill pure mechanical functions, but are also involved in complex signaling cascades determining intracellular processes and cell shape control [8–11]. Despite growing knowledge about relevant components of the podocyte cytoskeleton [5], we are far from a complete understanding how specific proteins determine podocyte function and morphology on a spatial and temporal level. In particular, it is less clear which and how molecular machineries stabilize podocyte FPs in health and disease.

Recent progress in ultrapure isolation procedures of podocytes provided now cell-specific and extensive expression information, both on a RNA as well as protein level [10,12–14]. Cross-analysis of these data with a focus on actin-bundling proteins like *alpha-Actinin-4* revealed a highly specific expression pattern for the EF-hand domain protein allograft inflammatory factor 1 like (AIF1L, other aliases are IBA2 or C9orf58—[15]). AIF1L is a homolog to the allograft inflammatory factor 1 (AIF1 or IBA1), sharing up to 60% sequence homology [15]. Previous work could demonstrate that AIF1L and AIF1 show actin bundling and cross-linking function, as well as co-localization and–sedimentation with F-actin [15]. More recently, increased expression levels for AIF1L were reported in cases of breast cancer and functionally related to increased proliferation rates via upregulation of cyclin D1 [16]. Nevertheless, the specific role and function of AIF1L other than influencing actin bundling remained unclear so far [14,15].

## Materials and methods

### Cell culture

Conditionally immortalized human podocytes were kindly provided by M. Saleem (University of Bristol, UK). Cells were cultured at 33°C in RPMI-1640 medium supplemented with 10% fetal calf serum (FCS), Penicillin/Streptomycin, ITS and non-essential amino acids. To induce differentiation, podocytes were cultured at 37°C for 10 to 14 days. Treatment experiments with the myosin-II inhibitor blebbistatin (Sigma, Germany) were performed on collagen IV coated Ibidi 8-well chamber slides (Ibidi, Germany) and 10μM blebbistatin in complete cell culture medium for 20 minutes. For blebbistatin washout experiments, podocytes were treated with 50μM blebbistatin for 1 hour, 3x washed with complete cell culture medium and cultured for another 20 minutes to allow reformation of the actomyosin cytoskeleton. Puromycin (PAN—InvivoGen, USA) treatment of differentiated podocytes was performed in complete cell culture medium with 3μg/ml PAN for 9 hour.

### Antibodies

All antibodies used in this study are collectively described in S1 Table.

### Immunofluorescence staining

Immunofluorescence staining of frozen kidney sections and cultured podocytes was performed as described previously [10]. In brief, podocytes were cultured overnight on collagen IV 8-well chamber slides (Ibidi, Germany) or glass coverslips coated with collagen IV (50ng/μl, Sigma, Germany). Cells were fixed in 4% paraformaldehyde in PBS for 10 minutes and permeabilized using 0.1% Triton X-100 in PBS. Permeabilized cells were blocked with 5% BSA in PBS for 1h at room temperature. Primary and secondary antibodies were diluted in blocking solution and incubated for 60 to 180 minutes or 45 minutes respectively. A Zeiss Axio Observer microscope or a LSM510 confocal microscope (Carl Zeiss, Oberkochen, Germany) equipped with 20x and 63x objectives were used for image acquisition. The Fiji NIH ImageJ 1.51 software was used to analyze whole cellular fluorescence intensities of Phalloidin (F-Actin) or p-MYL9 stained cells. Filopodia were analyzed on collagen IV 8-well chamber slides after staining for F-Actin by Phalloidin. The number of lamellipodium-based filopodia (length >0.25μm) was counted and the mean filopodia length per cell was measured using the Fiji NIH ImageJ 1.51 software. A similar staining protocol was used for immunofluorescence analysis of frozen kidney or paraffin embedded sections. For paraffin sections heat-induced antigen retrieval was performed in Tris-EDTA buffer at pH 9.0. Immunofluorescence stainings of human kidneys were performed on samples of unaffected areas of tumor nephrectomies as approved by the Scientific-Ethical Committee of the University Medical Center of Freiburg. Kidney samples of mice were obtained in accordance to the German law for the welfare of animals, the NIH Guide for the care and use of laboratory animals and were approved by the Regierungspräsidium Freiburg (TVA: G14/43).

### CRISPR/Cas9 mediated generation of *AIF1L* knockout clones

The CRISPR/Cas9 genome editing technology was used to generate *AIF1L* KO podocytes as described before [11]. gRNAs were designed targeting exon 4 or exon 5 of the human *AIF1L* gene (e-crisp.org—http://www.e-crisp.org/E-CRISP/) (gRNAs: 5’-GTGATTTCCAGAGAAGTACATGG-3’, 5’-GATGAAGAAGATGATCTCAGAGG-3’). This gRNAs were introduced in targeting vectors with an OFP reporter (gene-art, Invitrogen, Germany) according to manufacturer´s instructions. Targeting vectors were transfected by electroporation into immortalized human podocytes and OFP positive cells were subsequently selected via FACS sorting to generate isogenetic clones. The knockout of *AIF1L* was confirmed by Sanger sequencing and standard SDS-polyacrylamide gel electrophoresis based western blotting. Only clones with homozygous mutations resulting in premature stop-codons were used. Non-mutated clones were used as WT controls.

### Analysis of focal adhesion size and number

Measurement of focal adhesions was performed as described previously [17]. In brief, human immortalized podocytes were cultured on collagen IV coated glass coverslips and immunofluorescence staining was performed for the focal adhesion component PAXILLIN. Fluorescence images of stained cells were taken using a Zeiss Axio Observer microscope, equipped with a 63x objective and Apotome function. Analysis of focal adhesions was performed with a custom written macro embedded in the NIH Fiji ImageJ 1.51 software. Blebbistatin treated cells were cultured on collagen IV coated Ibidi 8-well chambers. Number of mature FA (>0.5μm^2^) per cell was manually measured and counted.

### Singe cell migration assay

Time lapse imaging was performed by using a Nikon Biostation IM device (Nikon, Düsseldorf, Germany). Podocytes were cultured on Ibidi tread μ-dish (Ibidi, Germany) for 24 hours before analysis and single cell migration was recorded for 12 hours. Analysis of time lapse data was done by using the ManualTracking and ChemoTaxis plugin implemented in the NIH ImageJ 1.46 software.

### Cell spreading on 3D collagen gels

3-D collagen gels were prepared by using bovine collagen I solution (PureCol, Advanced BioMatrix, USA) according to the manufacturer´s instructions. 20μl of diluted and pH adjusted working solution (0.3mg/ml collagen, pH 7.4) was spread to Ibidi glass bottom μ-dishes and polymerized at 37°C to create a thin collagen gel. *AIF1L* WT and KO podocytes were seeded and allowed to spread on top of these gels for 3 or 24 hours. Cells were stained and z-stack fluorescence images and 3D reconstructions were generated from podocytes after a spreading time of 3 hours. Fully spread and flattened cells were imaged by conventional 2D immunofluorescence after 24 hours.

### Live cell imaging and analysis of membrane dynamics

Live cell imaging of *AIF1L* knockout or wildtype podocyte was performed on Ibidi tread μ-dish (Ibidi, Martinsried, Germany). Phase contrast imaging was done by using a Zeiss Cell Observer microscope equipped with a LCI Plan-Neofluoar 63x/1.3 objective and Tokai Heat Incubator (controlled heating and CO_2_ atmosphere). The FIJI-ImageJ 1.51 software with the Multi-Kymograph tool was used to analyze cell membrane dynamics. For generation rate and persistence of filopodia only lamellipodium based mature filopodia were analyzed. The mean filopodia persistence per cell was calculated by tracking and analyzing 10 representative filopodia per cell. The number of newly formed filopodia per minute was counted to calculate the filopodia generation rate.

### Expression of plasmids in cultured podocytes

A full-length human AIF1L plasmid vector was obtained from OriGene (OriGene Technologies, Rockville, USA) and was subcloned into an EGFP-tagged pcDNA6 expression vector. For expression studies, 3–5μg DNA was transfected into podocytes by electroporation (Amaxa Nucleofector system, Lonza, Germany). Transfected cells were cultured for 48–72 hours before experimental analysis.

### Cell proliferation/MTT assay

Cell proliferation was analyzed using a commercial available MTT assay (Vybrant MTT Cell Proliferation Assay Kit, Thermo Fischer Scientific) according to the manufacturer´s instructions.

### Subcellular fractionation

The REAP method was used for subcellular fractionation of *AIF1L* WT and KO podocytes. A previously established protocol was essentially used and performed to obtain different subcellular fractions [18].

### Analysis of active Cdc42 levels

Cells were cultured in RPMI-1640 medium containing 1% FCS for 72 h. After serum starvation, cells were harvested and lysates were equalized due to protein content; Cdc42 activity was measured using a Cdc42 G-LISA activation assay kit according to manufacturer´s instructions (BK135, Cytoskeleton, USA).

### AIF1L immunoprecipitation and interaction proteomics

SILAC labeling and quantitative MS analysis of podocytes was performed as previously described [10,19]. *AIF1L* WT and KO podocytes were cultured on 15cm cell culture dishes to 90% confluency. Cells were lysed in Triton X-100 lysis buffer (1% Triton X-100, 20ml Tris-HCL, 50mM NaCL, 50mM NaF, 15mM Na_4_P_2_O_7_, 1mM EDTA, pH 7.4; for 30 min; 4°C) and centrifuged. Supernatants were balanced to a total amount of 4.3 mg protein per genotype and SILAC condition and incubated with 5μg of AIF1L antibody for 20 hours respectively. Thereafter lysates were incubated with 30μl of protein A-Sepharose beads for 90 minutes at 4°C. The beads were 5x repetitively centrifuged and washed with lysis buffer. Bound proteins were resolved in Laemmli sample buffer (95°C, 5 min) and processed for MS analysis or separated by standard SDS-polyacrylamide gel electrophoresis for western blotting.

For the SILAC-based MS analysis, AIF1L dependent precipitated proteins with a log2 fold enrichment of >0.4 in both replicates (compared to AIF1L KO as control) were defined as significantly enriched. The DAVID (version 6.8) analysis tool was used for GO-Term enrichment analysis of these AIF1L interactome (background: whole human proteome, significantly enriched GO-Terms: p-value<0.05 and count ≥ 2; [20,21]). The Cytoscape 3.6.0 software and the EnrichmentMap app were used for GO-Term visualization and network generation [22,23]. GO-Terms with an overlap of >2/3 of mapped proteins were assumed and visualized as connected. Heat maps were created using the GraphPad Prism software. For proteomic datasets and a detailed analysis see S1 Dataset.

Comparative in-vivo proteomic datasets of isolated mice podocytes were published earlier by Boerries M. et al. and Schell C. et al. and in this study reanalyzed for the podocyte specific enrichment of actin bundling proteins [10,12].

### Statistics and reproducibility

All statistical data were expressed as boxplots with whiskers from the 5th to 95th percentile. Paired Student´s t-test or ONE-WAY ANOVA test (multiple comparison analysis with Tukey`s post hoc test) were used based on data distribution. Statistical significance was defined as * p<0.05, ** p<0.01, *** p<0.001 and **** p<0.0001, n.s.—not significant. Numbers of independent experiments and total amount of analyzed cells are stated in the figure legends.

## Results

### AIF1L is specifically expressed in podocytes

**Fig 1:**
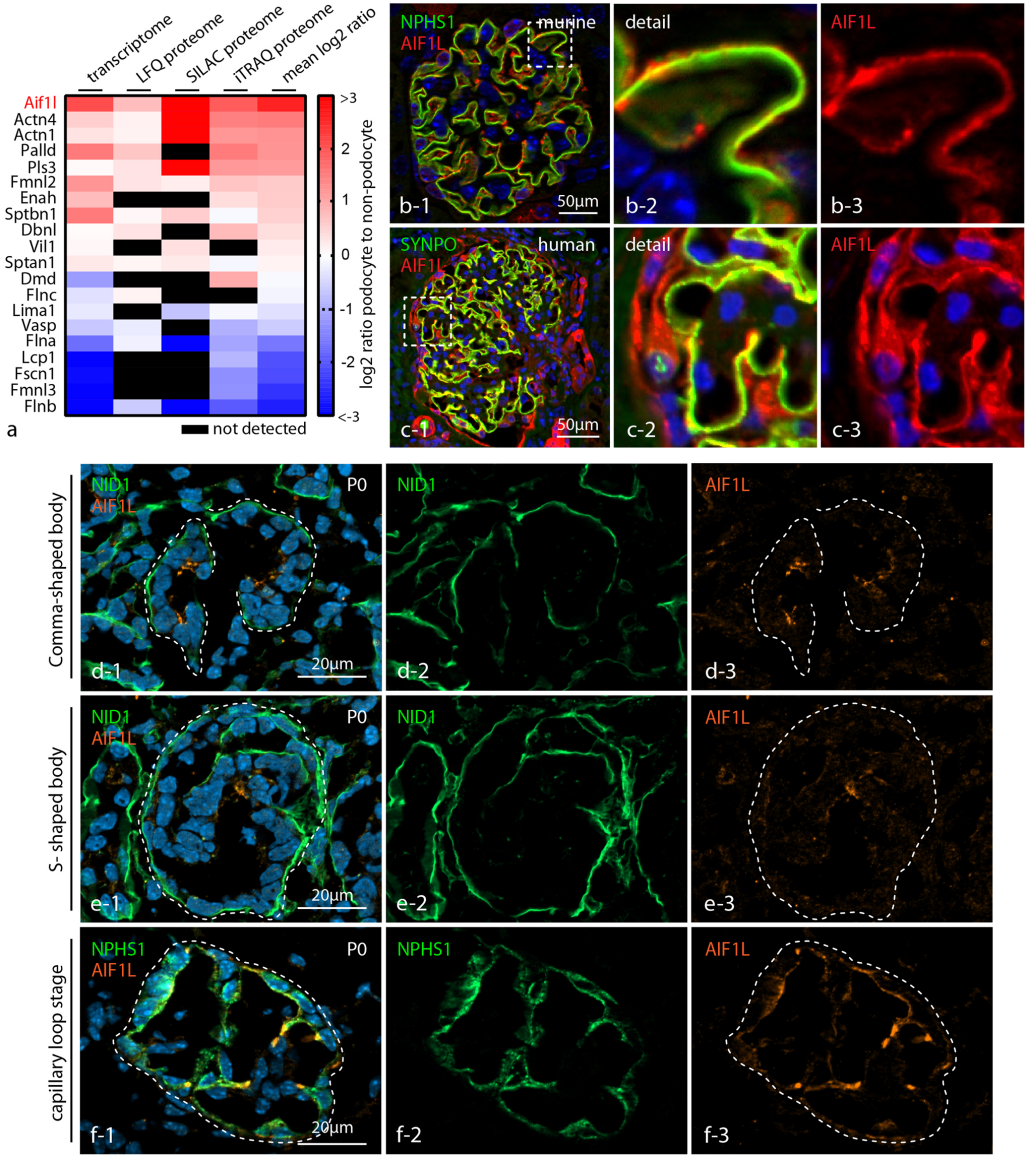
AIF1L is specifically expressed in podocytes. (a) Cross-analysis of transcriptome as well as proteome data sets for actin bundling proteins (unlabeled and quantitative—see for details Materials and methods sections) revealed highly selective expression of AIF1L in podocytes compared to non-podocyte glomerular cells. (b-c) Confocal immunofluorescence microscopy demonstrated pronounced co-localization between AIF1L and the podocyte compartment visualized by the specific slit diaphragm component NEPHRIN (NPHS1) or the podocyte specific cytoskeleton protein SYNPO (note expression of AIF1L also in tubular compartments and parietal epithelial glomerular cells in human kidney sections). Here, AIF1L localizes prominently to the basal compartment of podocytes as demonstrated by linear co-localization with NEPHRIN and SYNPO. Of note, AIF1L is also detectable in the whole podocyte cell (note divergences in cytoplasmic localization between mice and human podocytes). (d-f) Immunofluorescence microscopy on different glomerular maturation stages in a p0 wild type murine kidney section: AIF1L is present throughout all stages of glomerular maturation (Nidogen (NID1) was used as a marker for visualizing basement membrane structures, NPHS1 for the podocyte compartment).

Given the importance of actin-associated proteins for podocyte function [5,6], we filtered and re-analyzed transcriptome as well as proteome data sets derived from isolated (genetically-tagged) podocytes for actin crosslinking and bundling proteins [10,12]. Besides well described proteins such as the actin-crosslinker alpha-ACTININ-4 or the adhesion component PALLADIN [7,24], the highest overall expression was detected for Allograft inflammatory 1 like protein (AIF1L or IBA2 –Fig 1a). We employed confocal immunofluorescence microscopy to validate the selective expression of AIF1L within the podocyte compartment. In either human or murine glomerular sections we observed a distinct co-localization of AIF1L with the podocyte specific slit diaphragm protein NEPHRIN at the basal compartment of podocytes. In addition, also overlap between AIF1L and the cytoskeleton associated protein SYNPO was observed (Fig 1b and 1c). Aside from accumulation of AIF1L at the rather basal compartment, the protein was also detectable within the podocyte cell body (to a lesser extent).

Interestingly, AIF1L expression was not completely restricted to the glomerular compartment, but was also appreciated throughout the collecting duct (Fig 1c–S1 Fig). As podocytes undergo a drastic morphogenetic transformation during development characterized by progressive evolvement of basal specification and podocyte foot process formation, we wanted to identify at which developmental time point AIF1L is expressed. To this end we used immunofluorescence microscopy on murine kidney sections, where we detected expression of AIF1L from the early beginning of comma-shaped bodies and with increasing intensity at later developmental stages such as the capillary loop (Fig 1d–1f). Together these findings indicate that AIF1L shows a selective expression pattern within the glomerular compartment and is expressed from early beginning of podocyte differentiation.

### AIF1L localizes to filamentous actin, adhesion sites and cellular protrusions

**Fig 2:**
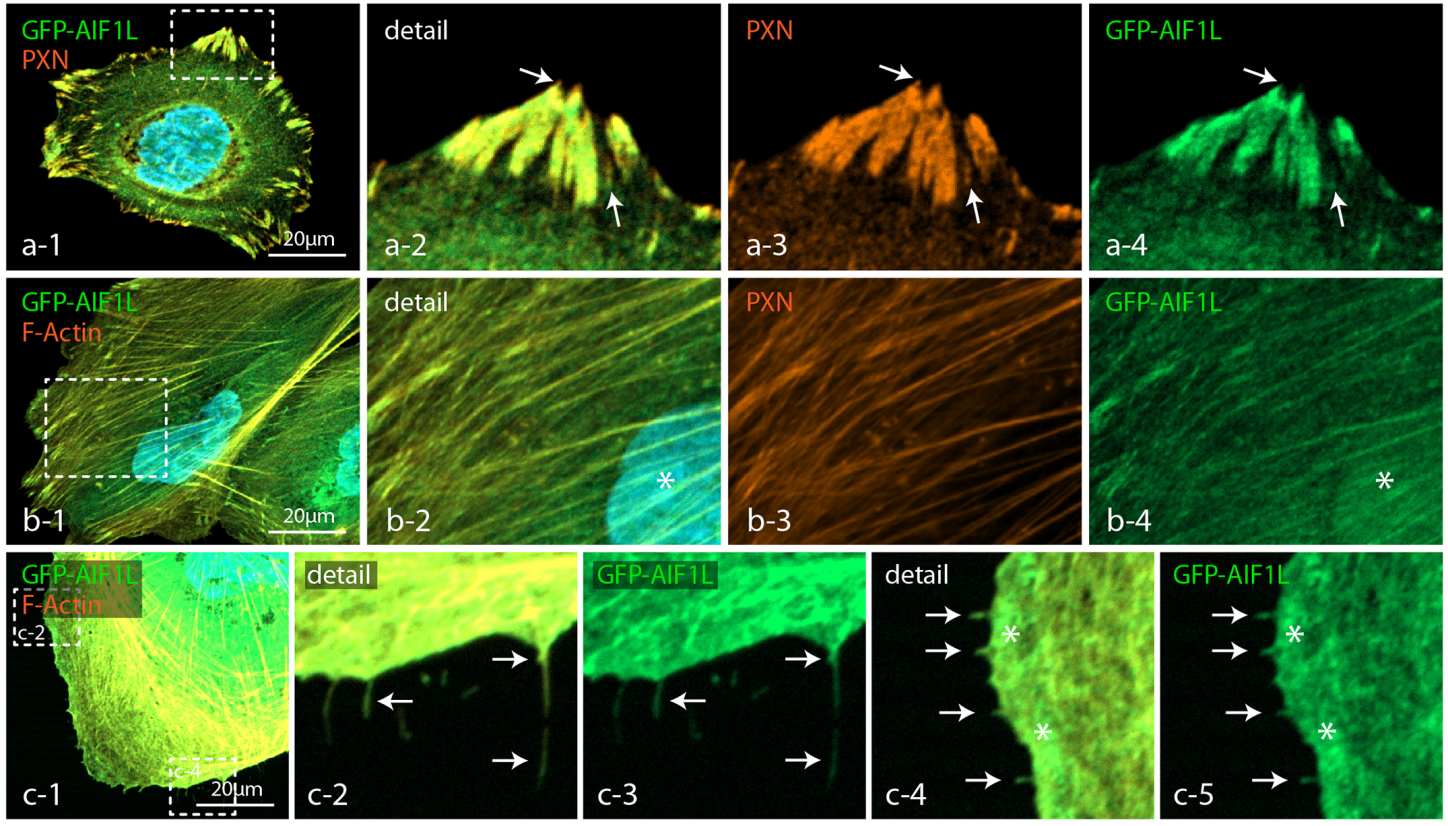
AIF1L localizes to filamentous actin, adhesion sites and cellular protrusions. (a) Expression of GFP-AIF1L in wild type podocytes localized to focal adhesion sites, as revealed by co-staining with the FA marker PAXILLIN (PXN—dashed boxes indicate sites of magnification, white arrows indicate FA tips or nascent FAs with less intense co-labeling of AIF1L and PAXILLIN). (b) In co-stainings with the filamentous actin marker phalloidin and the nuclear dye DAPI AIF1L localized to stress fibers and to the nuclear compartment (dashed boxes indicate sites of magnification; white asterisk indicates localization of AIF1L to the nucleus). (c) Filopodial extensions and the leading edge zone showed also an accumulation of AIF1L (white arrows indicate filopodial extensions; white asterisks indicate the leading edge zone; pictures in c were gamma and intensity adjusted to increase filopodia visualization).

To determine the specific subcellular localization of AIF1L we made use of GFP-tagged expression constructs and observed a predominant accumulation at mature focal adhesion (FAs) sites, showing co-localization with the bona fide FA protein Paxillin (Fig 2a). Furthermore, AIF1L was abundantly detected at ventral actin stress fibers (directly connecting to FA sites) as well as to a lesser extent in podocyte nuclei (Fig 2b and S1 Fig). Detailed analysis of AIF1L-expressing podocytes revealed extensive accumulation of AIF1L within discrete filopodial protrusions (Fig 2c). Filopodia are thin, actin-rich, finger-like membrane extensions, which predominantly evolve from pre-existing lamellipodia [25]. It is generally accepted that filopodia serve as cellular antennae for probing the extracellular environment and are involved in processes such as cellular migration [25].

### Loss of AIF1L does not impact cytoskeletal structure or migratory behavior

**Fig 3:**
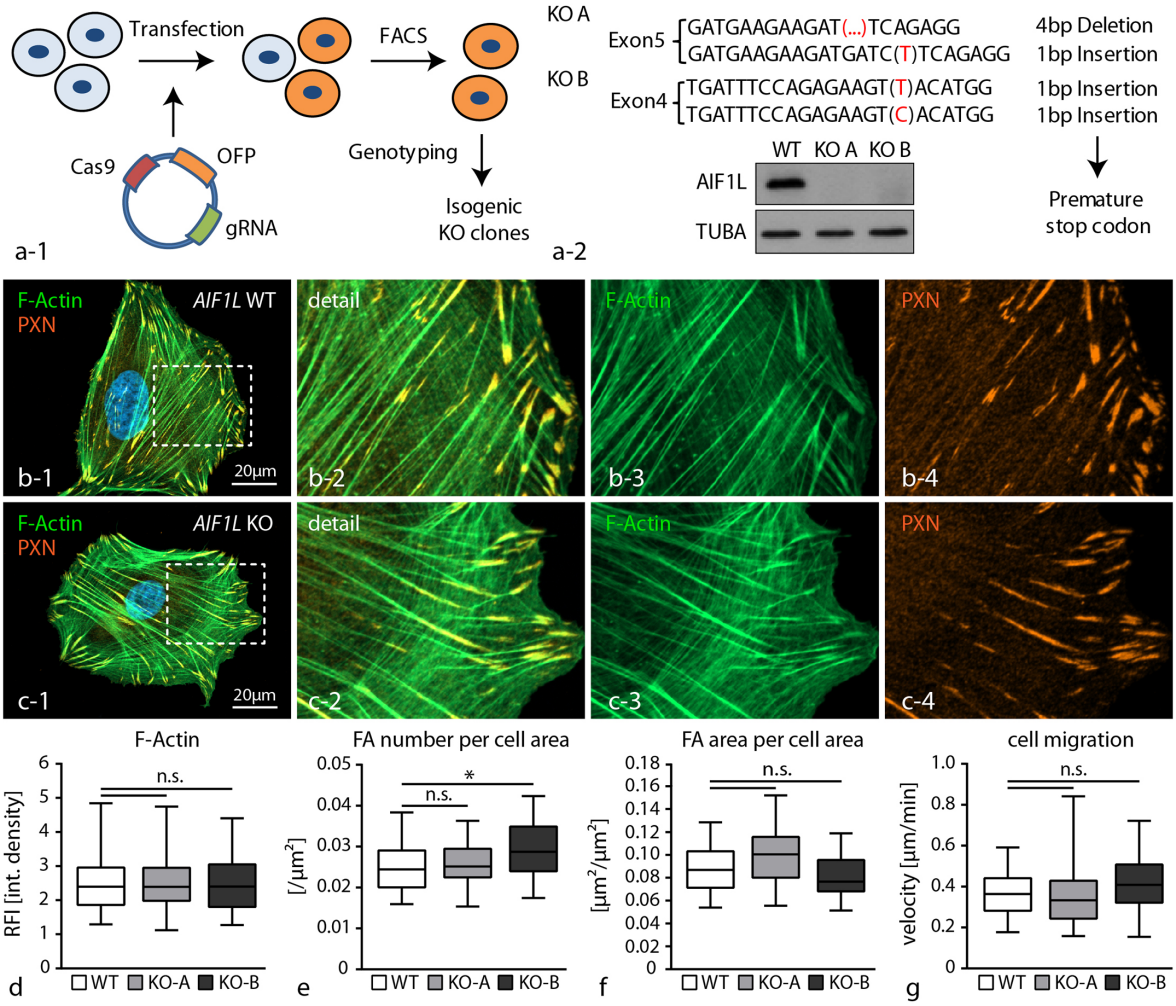
Loss of AIF1L does not impact cytoskeletal structure or migratory behavior. (a) Schematic depicting the generation strategy of *AIF1L* knockout clones using the CRISPR/Cas9 genome editing technology. After expression of a single vector encoding CAS9 protein as well as the gRNA and an OFP-reporter construct, single cell clones were generated and screened for NHEJ-repair events and consecutive occurrence of deleterious mutations. Sanger sequencings of two representative clones are included; western blot experiments confirmed loss of AIF1L protein in representative knockout clones (TUBA—tubulin alpha was used as a loading control). (b-f) Immunofluorescence stainings for FAs using PAXILLIN and for the F-actin cytoskeleton did not reveal major differences between knockout and wild type control cells. These observations were further corroborated by quantification for F-actin content and FA morphometry (n = 111 WT, 61 KO-A and 64 KO-B podocytes out of 3 independent experiments were analyzed for F-actin intensity; RFI—relative fluorescence intensity; n = 59 WT, 30 KO-A and 32 KO-B podocytes out of 3 independent experiments were analyzed for FA morphology; n.s.–non significant, * p<0.05). (g) Single cell migration experiments did not show any differences between wild type and AIF1L knockout cells (n = 135 WT, 145 KO-A and 92 KO-B podocytes out of 3 independent experiments were analyzed; n.s.–non significant).

In order to elucidate the cellular function of AIF1L in the context of podocytes, we employed a recently established genome editing workflow based on the CRISPR/Cas9 technology to generate complete knockout clones for the *AIF1L* locus (Fig 3a—[10]). Here, we used two different guide RNAs (sgRNAs) to target individual exons 4 and 5 within the *AIF1L* gene (Fig 3a). Based on screening of isogenetic clones we identified so far 5 complete knockout clones, which were qualitatively evaluated, confirmed by Sanger sequencing and western blot experiments (Fig 3a). For quantitative experimental analysis and data presentation one representative individual AIF1L knockout clone was selected for each sgRNA (targeting either exon 4 or exon 5). Given the predominant localization of AIF1L to the actin cytoskeleton and focal adhesions (FAs), we analyzed the morphology of respective knockout clones with special emphasis on cytoskeletal architecture and FAs. Here, morphological evaluation did not reveal any major alterations in terms of F-actin content, as well as number and size of FAs (Fig 3b–3f). These initial observations were furthermore corroborated by functional single cell migration assays, where AIF1L knockout clones showed identical migratory behavior as respective wild type controls (3g). These findings indicate that despite a clear association of AIF1L with F-actin rich stress fibers or FA sites, loss of AIF1L does not directly impact those structures, at least on a morphological level in steady state conditions.

### AIF1L prevents formation of filopodial extensions in podocytes

**Fig 4:**
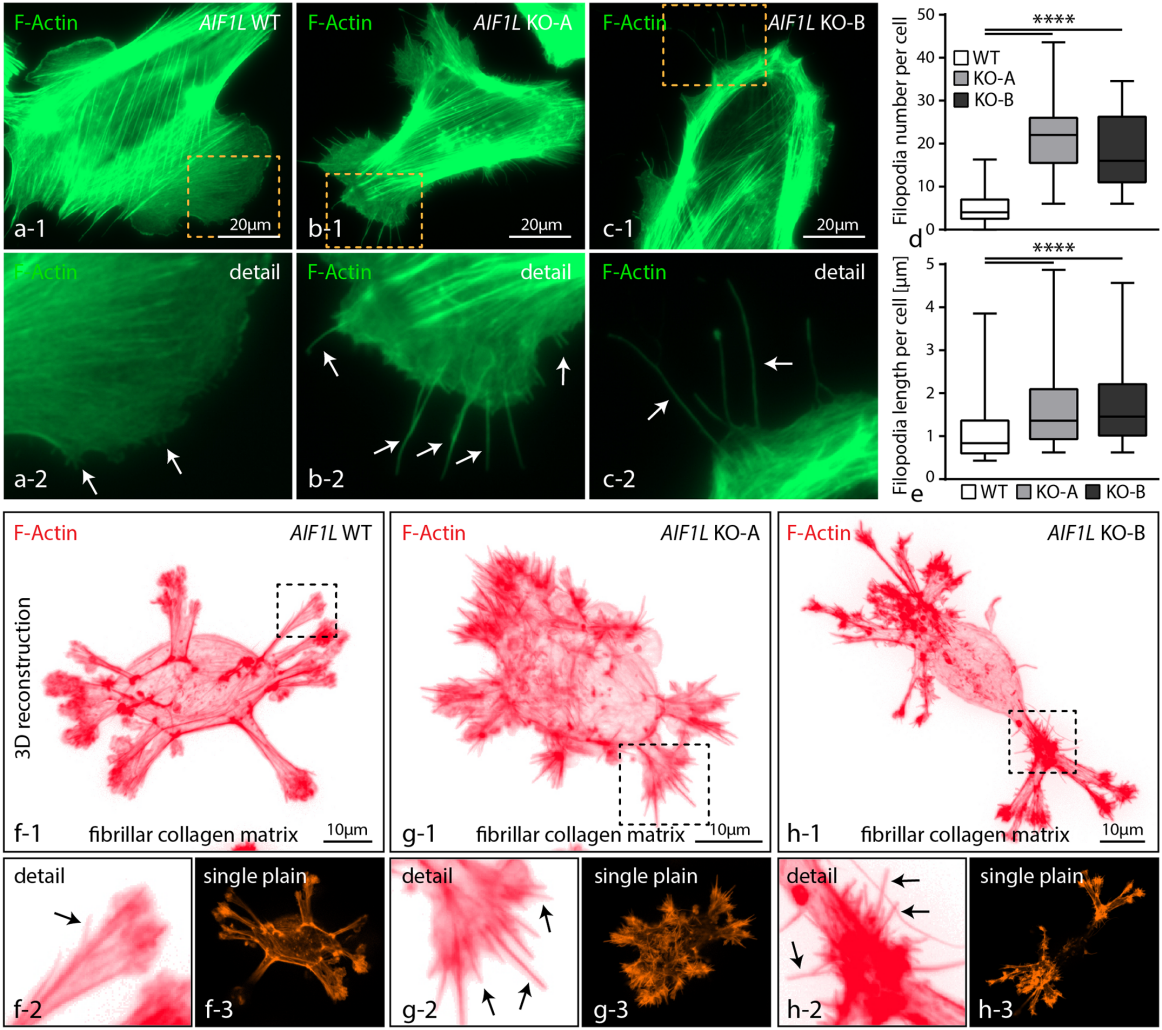
AIF1L prevents formation of filopodial extensions in podocytes. (a-c) Immunofluorescence studies revealed the presence of numerous thin filopodial extensions evolving prom pre-existing lamellipodial structures in both AIF1L knockouts (dashed boxes indicate areas of higher magnification; white arrows indicate filopodial extensions). (d-e) Quantification of filopodia showed higher numbers per cell in conditions of AIF1L loss; also, measurements of filopodia demonstrated overall increased length in respective AIF1L knockout clones (n = 53 WT, 53 KO-A and 42 KO-B podocytes out of 3 independent experiments were analyzed for filopodia number; filopodia from those cells were measured for length, n = 277 WT, 1175 KO-A and 778 KO-B filopodia; **** p<0.0001). (f-h) Seeding of podocytes on thin fibrillar collagen gels for 3 hours resulted in an overall multi-polar morphology with several membrane protrusions. Numerous filopodia extended from those areas of membrane protrusion in AIF1L knockout cells. (black arrows indicate filopodial extensions; dashed boxes indicate areas of magnification).

Our initial characterization of the subcellular localization pattern showed that AIF1L could also be detected at filopodial extensions (Fig 2c). Filopodia are thin, actin-based cellular protrusions, evolving from pre-existing lamellipodia [26]. Detailed analysis demonstrated that loss of AIF1L resulted in the formation of numerous filopodial extensions in respective knockout clones (Fig 4a–4c). Further quantification confirmed not only increased numbers of filopodia in AIF1L knockout clones, but also showed that loss of AIF1L led to an overall increase of filopodial length (Fig 4d and 4e). It is well known that morphological and functional features are essentially influenced by environmental cues such as matrix rigidity or ECM ligand composition [27]. Podocytes reside on a highly specialized basement membrane (glomerular basement membrane—GBM), which is mainly composed of collagen type IV [28]). In order to test whether the morphological phenotype of AIF1L knockout cells is influenced by environmental parameters, we seeded respective wild type and knockout cells on fibrillar collagen matrices. Here, we observed a multipolar morphology of wild type as well as knockout cells characterized by the generation of several protrusion fronts (Fig 4f–4h). AIF1L knockout cells generated a multitude of filopodial extensions evolving from those protrusion sites, whereas respective wild type control cells showed this feature only very infrequently (Fig 4f–4h and S2 Fig). Based on these observations, AIF1L appears to prevent the formation and generation of filopodial extensions in podocytes.

### Loss of AIF1L facilitates filopodia generation by modulating cell membrane dynamics

**Fig 5:**
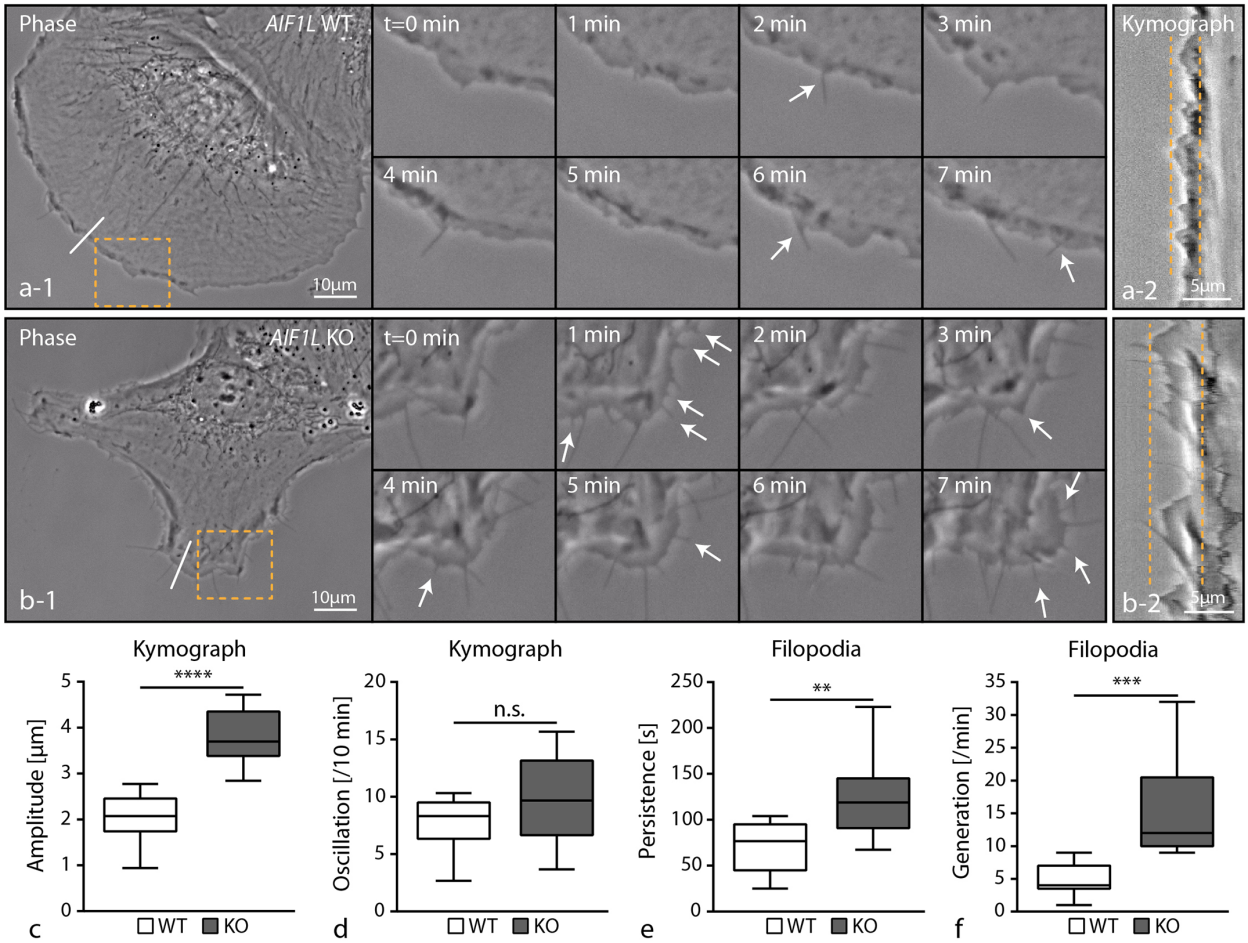
Loss of AIF1L facilitates filopodia generation by modulating cell membrane dynamics. (a-b) Live cell imaging, using phase-contrast microscopy, visualized the highly abundant generation of new filopodial extensions in AIF1L knockout clones. The leading edge in respective knockout clones showed furthermore an increased oscillation rate as a parameter of altered membrane dynamics (dashed boxes indicate areas of magnification; white arrows indicate individual filopodial extensions; dashed lines in a-2 and b-2 mark the maximal oscillation of the leading edge in kymographs corresponding to the white line in cells, a-1 and b-1). (c-d) Quantification of kymographic data revealed higher amplitude of membrane protrusions in AIF1L knockout cells and a tendency towards a higher oscillation rate (n = 9 representative WT and KO podocytes were analyzed; n.s.–non significant; **** p<0.0001). (e-f) Filopodia in AIF11L knockout clones showed a higher persistence and generation rate when compared to control wild type cells (filopodia of n = 9 representative WT and KO podocytes were analyzed; ** p<0.01; *** p<0.001).

We employed live imaging microscopy to characterize the nature of filopodia in AIF1L knockout podocytes. As filopodia commonly emerge from pre-existing lamellipodia, we performed kymographic analysis of the leading edge and recognized highly dynamic membrane oscillations in AIF1L knockout clones (Fig 5a–5d). These observations indicate that AIF1L might act as a stabilizing factor of the leading edge and that loss of AIF1L conversely results in increased cortical membrane turnover conditions. Analysis of individual mature filopodia revealed that AIF1L loss results in an increased persistence and generation rate reflecting our initial observations of increased filopodial numbers in AIF1L knockout clones (Figs 5e, 5f and 4d and S1 and S2 Movies).

### AIF1L interacts with components of the actomyosin machinery and nuclear proteins

**Fig 6:**
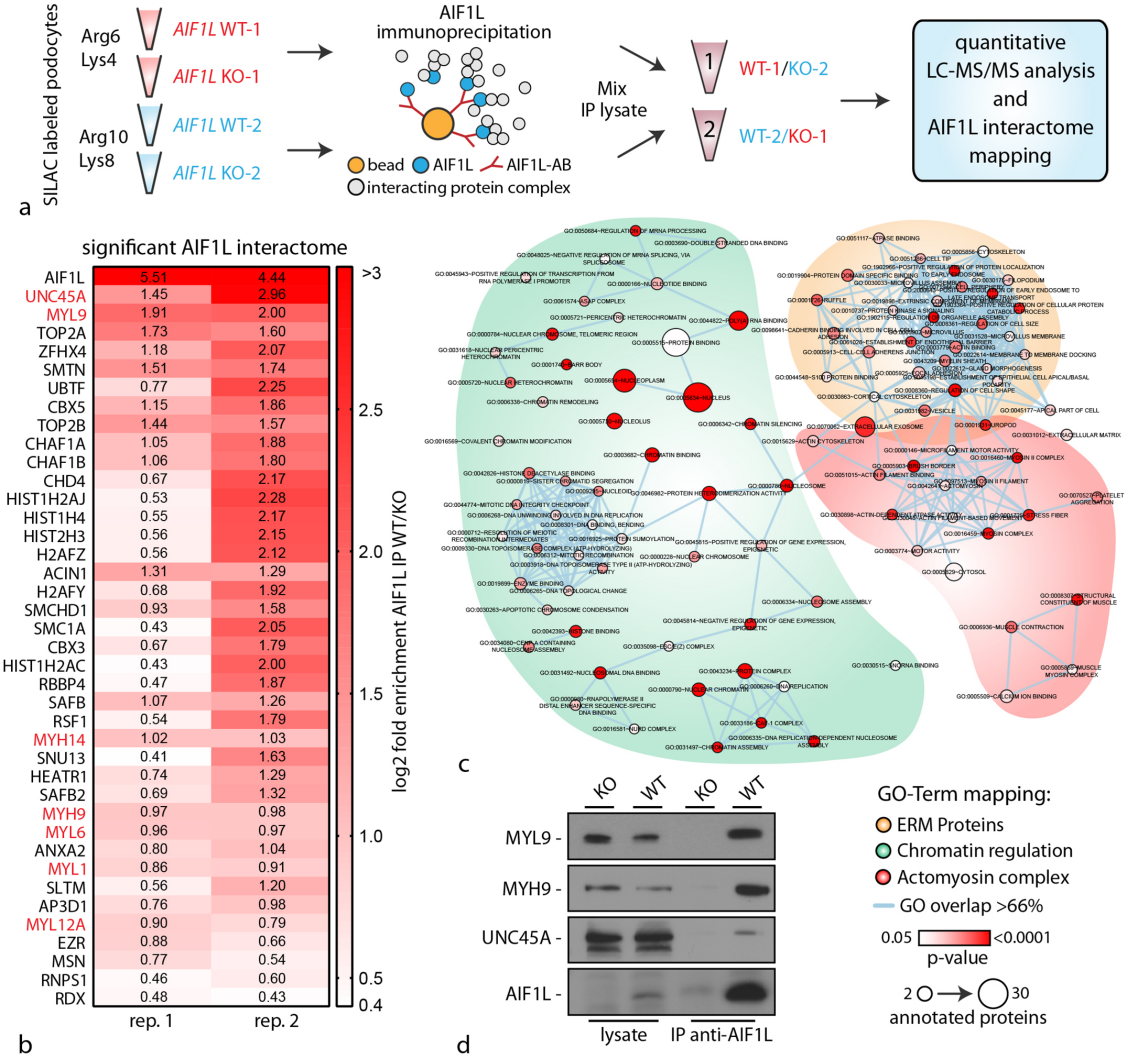
AIF1L interacts with components of the actomyosin machinery and nuclear proteins. (a) Schematic depicting the labeling strategy of wild type and AIF1L knockout clones employing the SILAC technology, and the consecutive immunoprecipitation as well as sample preparation for analysis via mass spectrometry. (b) Color-coded tabular presentation of proteins according to their enrichment score (normalized to the AIF1L knockout control) in two replicates. Red colored protein names belong to the non-muscle myosin-II actomyosin machinery. (c) GO-Term mapping of interaction partners from the knockout controlled immunoprecipitation experiments. Aside from proteins belonging to the actomyosin machinery, also ERM proteins and chromatin regulating proteins were mainly detected. (d) Validation experiments employing western blot confirm the association of MYL9, MYH9 and the chaperone UNC45A with AIF1L.

In contrast to its homologue AIF1, knowledge concerning AIF1L is only sparse. To better understand the functional role of AIF1L, we employed quantitative, knockout controlled, SILAC-based interaction proteomics. Therefore wild type and AIF1L knockout cells were efficiently labeled with stable isotope amino acids (SILAC—stable isotope labeling with amino acids, Fig 6a) and immunoprecipitation was performed using knockout validated AIF1L antibody. Wildtype and knockout IP Lysates were comparatively analyzed using mass spectrometry (Fig 6a). In two technical replicates we detected AIF1L as the protein with the highest enrichment score, directly followed by the myosin chaperone UNC45 and the regulatory light chain of the non-muscle myosin-II complex MYL9 (Fig 6b). Further analysis revealed that AIF1L precipitated various members of the non-muscle myosin-II complex machinery such as MYH14, MYH9, MYL6 and MYL12a (Fig 6b). In addition, GO-TERM mapping demonstrated that aside from members of the actomyosin machinery, ERM proteins as well as several nuclear proteins involved in chromatin regulation showed potential interaction with AIF1L (Fig 6c). The latter finding is in line with our observations that AIF1L showed not only a subcellular localization at actin stress fibers and FA sites, but also a distinct accumulation within the nucleus (Fig 2b and S1 Fig). Furthermore, we could demonstrate by subcellular fractionation experiments that AIF1L can be detected in the nuclear compartment (S3 Fig). Given the highest enrichment scores for UNC45 and MYL9, we furthermore validated these interactions by performing immunoprecipitation experiments corroborating our proteomic studies (Fig 6d).

### AIF1L knockout clones exhibit an impaired actomyosin contractility reserve

**Fig 7:**
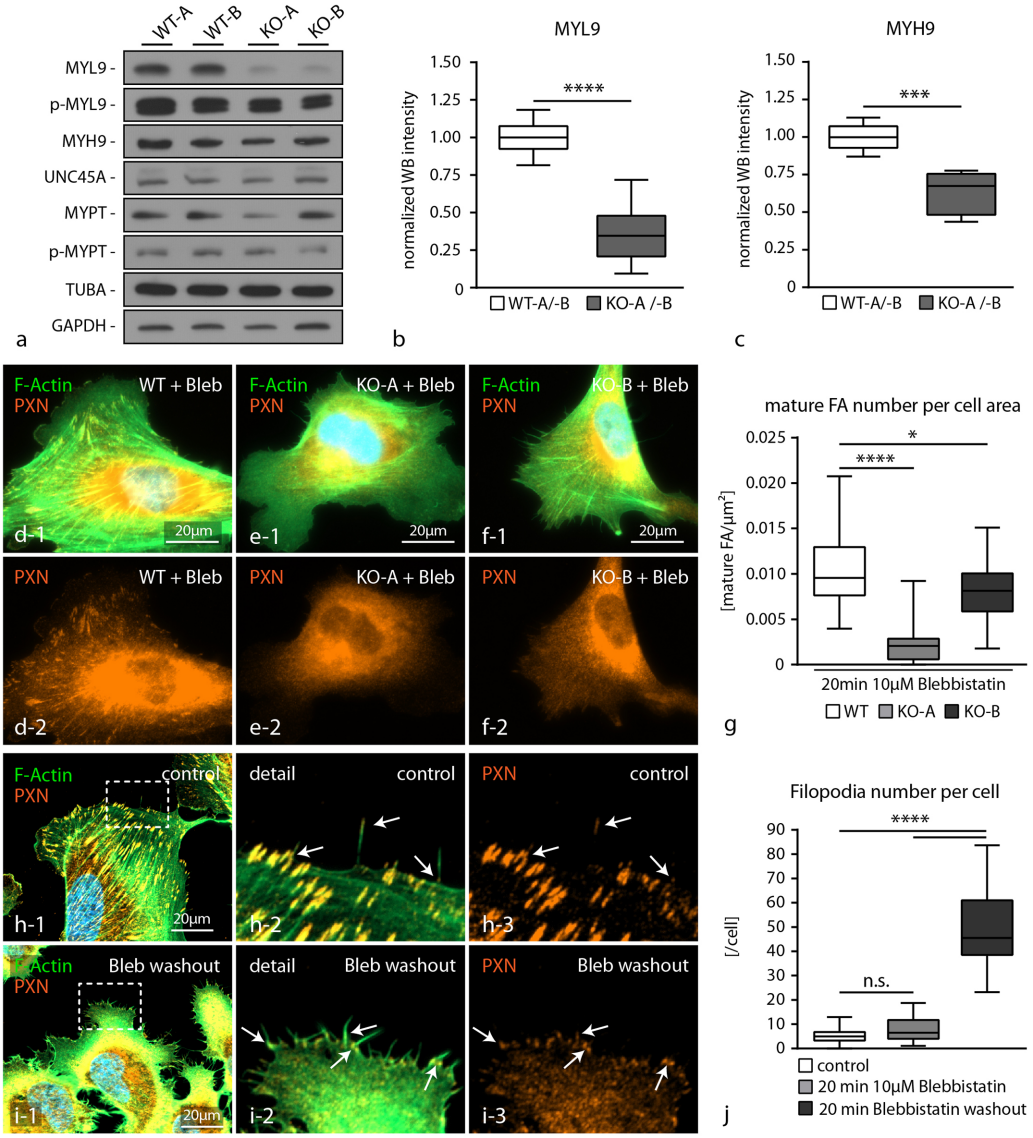
AIF1L knockout clones exhibit an impaired actomyosin contractility reserve. (a-c) Western blot experiments and quantification by densitometry demonstrated decreased levels of MYL9 as well as MYH9 in respective AIF1L knockout clones, whereas UNC45A or MYPT were not affected (n = 6 WT and KO WB intensities out of 3 independent experiments; *** p<0.001; **** p<0.0001). (d-g) Treatment of wild type cells and AIF1L knockout clones with the myosin-II inhibitor blebbistatin resulted in a more rapid dissolution of FA complexes in conditions of AIF1L loss, indicating a decreased actomyosin contractility reserve (n = 29 WT, 27 KO-A and 29 KO-B podocytes out of 3 independent experiments were analyzed; * p<0.05; **** p<0.0001). (h-j) Washout experiments for blebbistatin showed that in conditions of reconstituting actomyosin contractility podocytes show a high generation rate of filopodial protrusions (note that these structures are associated with FA sites at the filopodial basis indicated by white arrows; white dashed boxes indicate areas of magnification;(n = 20 podocytes per condition were analyzed; n.s.–non significant, **** p<0.0001)).

It is well known that the activity of the actomyosin machinery is directly influencing cell morphology and shape [29]. We reasoned that the pronounced generation of filopodia in AIF1L knockout cells and the interaction between AIF1L and the actomyosin machinery might attribute to this phenotype. Therefore, we evaluated the protein and phospho-protein levels of several core actomyosin machinery proteins in AIF1L knockout clones. Here, we observed a pronounced decrease in total levels of myosin-regulatory light chain (MYL9) as well as myosin heavy chain (MYH9). Of note, although total levels of MYL9 were drastically reduced in AIF1L knockout cells, the level of phospho-MYL9 was only modestly impaired (Fig 7a–7c and S4 Fig). Next, we wanted to functionally test whether the reduced levels of MYL9 and MYH9 might also be reflected by a reduced contractile capacity of the actomyosin machinery. To this end, we employed the myosin-II inhibitor blebbistatin and observed here reduced numbers of mature FAs in both knockout clones when compared to wild type control cells (Fig 7d–7g). Of note, FA number and area were not altered in AIF1L knockout clones under steady state conditions (Fig 3). This observation of an increased sensitivity towards actomyosin inhibition by blebbistatin thereby reflects the impaired contractile capacity in conditions of AIF1L loss. Finally, we were wondering how impaired actomyosin contractility and the generation of numerous filopodia in AIF1L knockout clones might be interconnected. Filopodia are characterized by a specialized focal adhesion machinery commonly termed as filopodial focal complexes (FX), which consist of a series of bona fide FA proteins and are thought to be involved in the generation of filopodial as well as lamellipodial contractility [30]. In fact, FXs could easily be visualized using immunofluorescence staining for the FA component paxillin in wild type cells at the filopodial tip (Fig 7h). Interestingly, inhibition of myosin contractility via blebbistatin treatment did not result in altered numbers of filopodia in wild type cells. However, we observed in blebbistatin washout experiments (reflecting a state of reconstituting actomyosin contractility) a pronounced formation of filopodial extensions, which were accompanied by FA formation at the filopodial base (Fig 7h–7j). These findings support the notion that a slight imbalance in actomyosin contractility, as seen in AIF1L knockout cells, might be involved in the generation of filopodial extensions.

## Discussion

There is a growing body of evidence based on genetic as well as experimental studies demonstrating that podocytes rely on a highly specialized cytoskeletal apparatus in order to establish and maintain the integrity of the kidney filtration barrier [5,6]. Context dependent requirements are also reflected by a cell type specific composition of the cytoskeleton, as demonstrated by others and us (e.g. SYNPO, ARHGAP24 or CORO2B –[17,31,32]). Proteomic analysis for selectively or highly enriched expression of cytoskeletal proteins led to the identification of AIF1L in glomerular epithelial cells (Fig 1). In fact, AIF1L (allograft inflammatory factor 1 like) is characterized by the presence of a calcium binding EF-hand domain and shares high homology to its homologue AIF1 [15]. Interestingly, a previous study identified AIF1 also to be expressed as a constitutive protein in human and rat podocytes based on DNA microarray studies [33]. These authors could also show a pronounced upregulation of AIF1 in the glomerular compartment in an inflammatory model (anti-GBM nephritis model), although the main source was identified to be intruding inflammatory cells [33].

AIF1 was initially cloned and identified in human as well as rat macrophages from heart transplants with chronic transplant rejection [34,35]. Further work could demonstrate that IFNy as well as other inflammatory cytokines can induce AIF1 mRNA levels in smooth muscle cells, whereas expression is constitutive in lymphoid tissues (e.g. spleen–[36]). Based on high expression levels within mononuclear cell lineages, AIF1 is used as a differentiation marker for macrophages or microglia [37]. An extensive body of work could demonstrate the involvement of AIF1 in various disease conditions ranging from rheumatoid arthritis [38], systemic sclerosis [39], encephalomyelitis [40] to cancer [41]. On a molecular level it was shown that AIF1 influences migration, cytokine secretion and proliferation thereby modulating disease conditions [42,43].

Conversely, aside from molecular homology and actin-bundling function only very little is known about expression and functional implications of AIF1L. Only very recently, it was shown that AIF1L is highly expressed in conditions of breast cancer and correlates with proliferation behavior via modulation of cyclin-D1 levels [16]. These findings are in accordance with previous reports about the role of AIF1 in cancer and breast cancer in particular [41,44,45]. In the context of podocytes, a recent study identified *Aif1l* with several other genes highly enriched in murine podocytes using single cell RNA-Seq approaches [14]. Further validation experiments employing siRNA knockdown showed modest effects on the F-actin content of murine podocytes [14]. Other than that mechanistic studies on the functional role and impact of AIF1L were missing so far. Here, we generated a series of complete knockout clones for AIF1L in human immortalized podocytes using CRISPR/Cas9 genome editing technology (Fig 3). In contrast to the aforementioned study we did not appreciate obvious effects on the actin cytoskeleton or F-actin content in knockout podocytes (Fig 3), even though it is documented that AIF1L exerts F-actin bundling activity [15]. This discrepancy might be explained by different approaches to modulate gene dosage, either by acute reduction employing siRNA or constitutive deletion by using CRISPR/Cas9 genome engineering. In fact, the latter approach might result in compensatory events (e.g. loss of AIF1L might be outbalanced by other actin cross-linking proteins or AIF1) as it was reported recently for zebrafish mutants and morphants (morpholino knockdown–[46]).

Given the F-actin bundling activity and co-sedimentation with filamentous actin, AIF1L shows a distinct co-localization with actin stress fibers (Fig 2). Moreover, we could also show a pronounced accumulation at focal adhesion sites, the nuclear compartment and filopodial extensions (Fig 2 and S1 Fig). While we did not observe a major impact on focal adhesion morphology under steady state conditions nor drastic alterations of migratory behavior in AIF1L knockout clones, we detected a profound increase in numbers of filopodial extensions (Figs 3 and 4). Filopodia are thin, elongated membrane protrusions which consist of linear actin bundles and are accepted as cellular antennae involved in extracellular sensing, probing and efficient migration [25]. In contrast to lamellipodial protrusions which are mainly based on Arp2/3 dependent propulsive branched actin-networks, filopodia are composed of paralleled bundles of F-actin [25]. In a series of previous studies a plethora of proteins has been identified to be involved in the generation of filopodial membrane protrusions ranging from small GTPases such as Cdc42 to actin-bundling proteins such as fascin [25]. Remarkably, we did not detect any significant alterations in the activation level of CDC42 in respective AIF1L knockout clones, pointing towards an alternative mode of filopodia generation (S4 Fig).

Currently two major theoretical concepts exist trying to explain and dissect the molecular process of filopodia formation, namely the convergent elongation model (reorganization of pre-existing Arp2/3 dependent actin filaments into filopodia) and the de novo filament nucleation model (formin dependent nucleation of actin filaments, independently of the Arp2/3 complex). However, further studies demonstrated that the relative contribution of the aforementioned models might heavily depend on cell-type specific properties and contextual as well as environmental cues [25,47].

Podocytes exhibit a highly complex cytological architecture, consisting of primary and secondary processes, which are required to attach to glomerular capillaries [48]. Simplification and retraction of these delicate secondary processes is the hallmark of any podocyte pathology (termed as foot process effacement–[48]). As the underlying mechanism increased and dysregulated membrane dynamics were identified [32,49]. In this context, increased membrane oscillations and exuberant formation of filopodia in AIF1L knockout podocytes might indicate the requirement of AIF1L for podocyte cytoskeletal homeostasis (Figs 4 and 5). And indeed, also other, podocyte specific, bona-fide cytoskeletal proteins have been previously implicated in the prevention of filopodia formation [50,51], supporting the concept that dysbalanced filopodia generation might serve as surrogate marker for stabilized cortical cytoskeleton and podocyte morphology [52,53]. Based on our knockout controlled, quantitative interaction proteomics approach we identified several members of the myosin-family as AIF1L binding partners and significant reduction in total protein levels of those (e.g. MYL9 and MYH9 –Fig 6). Interestingly, UNC45 as an established chaperone for myosin proteins was also detected as an AIF1L interacting protein (Fig 6 –[54]). At this point, one can only speculate how loss of AIF1L results in this alteration of the actomyosin machinery which translates also to a decreased contractile reserve (Fig 7). One potential explanation could be that AIF1L modulates folding or localization properties of UNC45 which are finally required for myosin maturation and assembly [55]. The absence of obvious alterations of focal adhesion morphology or direct effects on the cytoskeleton can potentially be explained by compensatory events such as stabilization of MYL9 activation levels (see also Figs 3 and 7). To this end, we can only exclude a direct effect of AIF1L on the CDC42 signaling axis as a cause for filopodia formation (see also S4 Fig). Since data on a AIF1L knockout model are not available yet, it is difficult to predict the precise in vivo role for AIF1L. To date, there are only studies available focusing on the role of the homologue AIF1 (Casimiro et al., 2013; Chinassamy et al., 2015; Kishikawa et al.; 2017). Remarkably, aside from effects on autoimmunity there are no data reporting about a significant physiological role of AIF1 for glomerular function. Given the high similarity of AIF1 and AIF1L one could hypothesize that only combinatorial knockouts could achieve meaningful insights in this context. The *in vivo* analysis of AIF1L (and AIF1) in the context of glomerular function definitely requires more work in the future.

In summary, we identified the actin-bundling protein AIF1L as a highly enriched protein in human podocytes, which localized to stress fibers, FAs and to a lesser extent also in the nuclear compartment. Our interaction proteomics identified several members of the non-muscle actomyosin machinery repertoire and furthermore revealed that loss of AIF1L results in a decreased actomyosin contractility reserve. Finally, we demonstrate that AIF1L is required to prevent the generation of filopodia and stabilizes podocyte membrane dynamics which might translate to its potential in vivo role via maintaining podocyte foot process morphology.

## Supporting information

S1 Dataset AIF1L interaction proteome. (XLSX)

S1 Fig Fluorescence microscopy of AIF1L protein expression and localization. (a) Immunofluorescence staining on murine kidney sections demonstrated a selective expression of AIF1L within the glomerular compartment as well as in proximal tubules of the cortex. The podocyte compartment was stained by NPHS1 (b) Expression of GFP-AIF1L showed aside from localization of AIF1L towards the filamentous actin cytoskeleton and focal adhesion sites, also accumulation of AIF1L in nuclei of podocytes (white asterisks indicate nuclei).
(JPG)

S2 Fig Loss of AIF1L cause a pronounced formation of filopodial protrusions. (a-c) Staining of the filamentous actin cytoskeleton in wild type and AIF1L knockout cells demonstrated pronounced formation of filopodial protrusions in respective knockout clones (dashed boxes indicate areas of magnification; white arrows indicate filopodia; pictures were gamma adjusted to increase filopodia visualization). (d-f) Seeding of podocytes on fibrillar collagen for 24 hours resulted in AIF1L knockout clones in the formation of numerous filopodia extensions (white arrows indicate filopodia; pictures were gamma adjusted to increase filopodia visualization).
(JPG)

S3 Fig AIF1L localizes to the nuclear compartment and regulate podocyte stress response. (a) Subcellular fractionation showed aside from cytoplasmic presence of AIF1L, also distinct accumulation in the nuclear compartment. The cytoplasm marker TUBA and the nuclear marker HIST1H3A demonstrate successful fractionation of these compartments. (b) Western blot for protein levels of cyclin-D1 revealed no major differences between wild type and respective AIF1L knockout clones. (c) Cell proliferation was assessed employing the MTT assay; here uniform differences in terms of proliferation were not detected (n = 3 independent experiments). (d-e) Evaluation of levels of cleaved-Caspase 3 demonstrated that knockout clones showed a lower level of accumulation of cleaved-Caspase 3 upon treatment of podocytes with the podocyte toxic agent puromycin aminonucleoside (PAN) (n = 6 WT and KO western blot (WB) intensities out of 3 independent experiments; **** p<0.0001).
(JPG)

S4 Fig Immunofluorescence evaluation of pp-MYL9 levels in AIF1L knockout podocytes. (a-c) Immunofluorescence evaluation of levels for pp-MYL9 demonstrated lower levels in respective AIF1L knockout clones when compared to wild type control cells (n = 110 WT, 61 KO-A and 64 KO-B podocytes were analyzed; **** p<0.0001). (d) Analysis of CDC42 activity by ELISA measurements could not detect any significant differences between levels of GTP-CDC42 between wild type and knockout cells (3 WT, 2 KO-A and 2 KO-B samples were analyzed; n.s—non significant).
(JPG)

S1 Movie AIF1L WT podocyte. (AVI)

S2 Movie AIF1L KO podocyte. (AVI)

S1 Table List of antibodies used in this study. (XLSX)
