# Supplementary figures and images for: AIF1L regulates actomyosin contractility and filopodial extensions in human podocytes
Source: PLoS One. 2018 Jul 12;13(7):e0200487. doi: 10.1371/journal.pone.0200487 (PMC6042786; doi:10.1371/journal.pone.0200487)

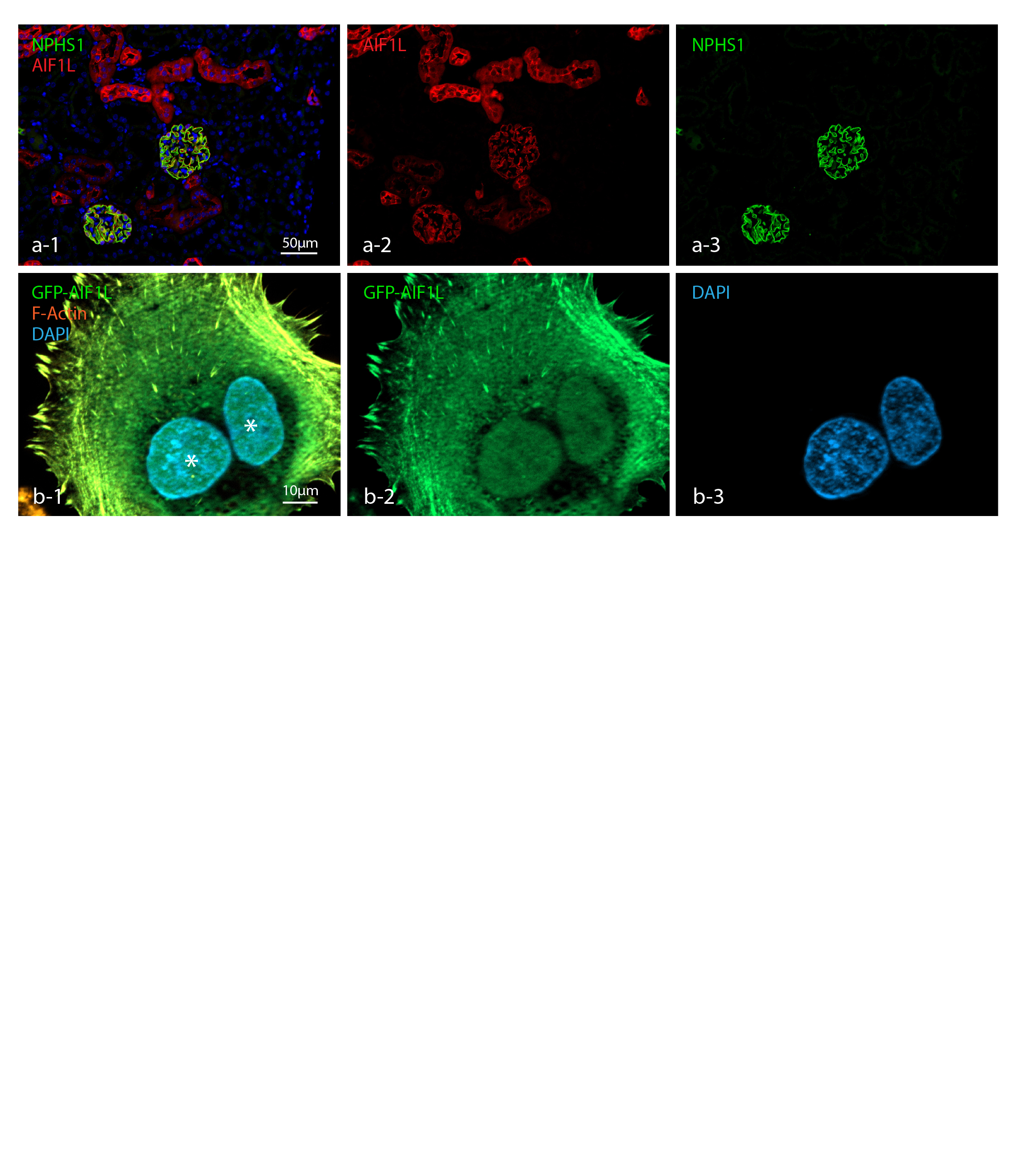

Supplement: S1 Fig — (a) Immunofluorescence staining on murine kidney sections demonstrated a selective expression of AIF1L within the glomerular compartment as well as in proximal tubules of the cortex. The podocyte compartment was stained by NPHS1 (b) Expression of GFP-AIF1L showed aside from localization of AIF1L towards the filamentous actin cytoskeleton and focal adhesion sites, also accumulation of AIF1L in nuclei of podocytes (white asterisks indicate nuclei). (JPG) [file pone.0200487.s002.jpg]

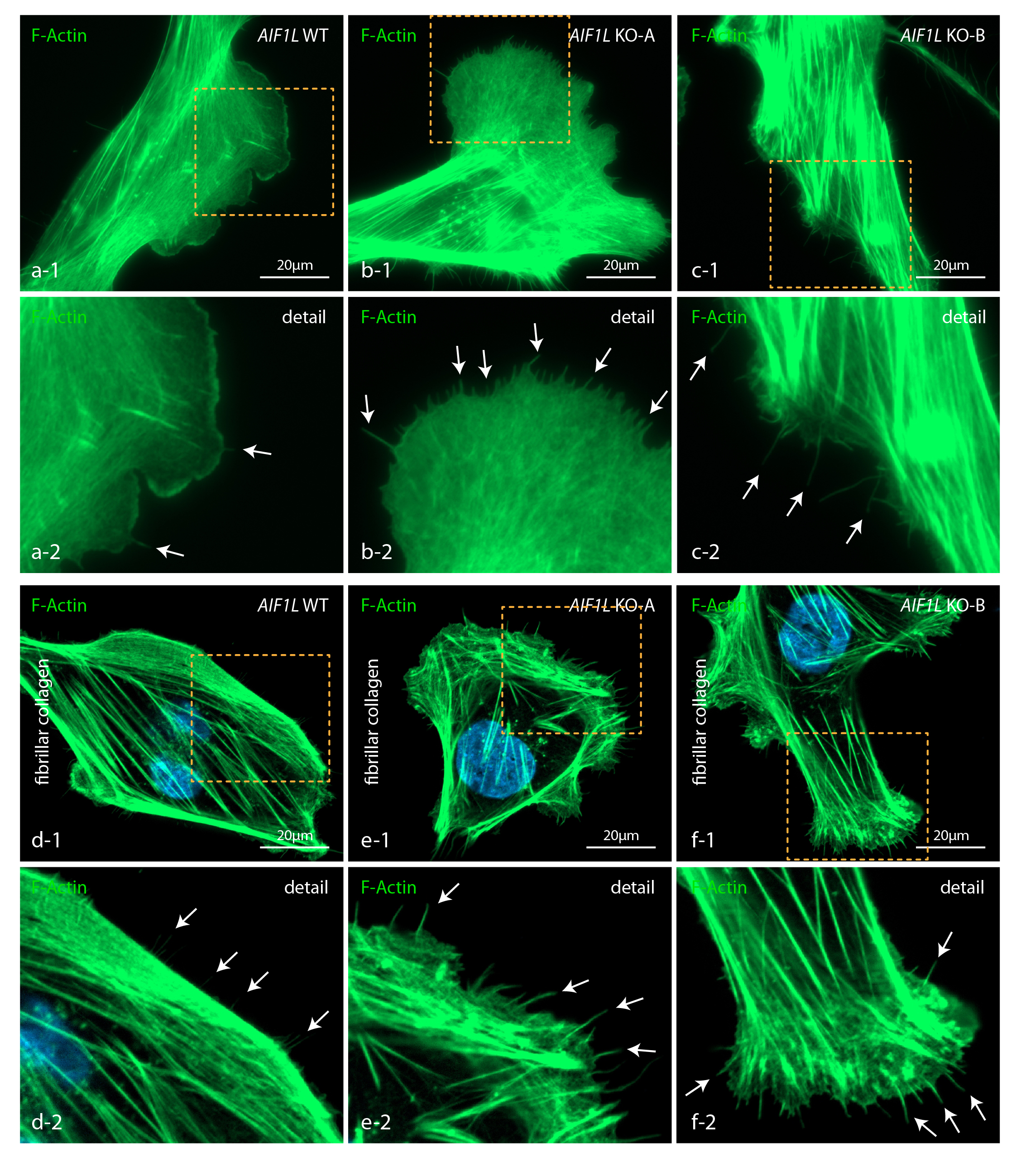

Supplement: S2 Fig — (a-c) Staining of the filamentous actin cytoskeleton in wild type and AIF1L knockout cells demonstrated pronounced formation of filopodial protrusions in respective knockout clones (dashed boxes indicate areas of magnification; white arrows indicate filopodia; pictures were gamma adjusted to increase filopodia visualization). (d-f) Seeding of podocytes on fibrillar collagen for 24 hours resulted in AIF1L knockout clones in the formation of numerous filopodia extensions (white arrows indicate filopodia; pictures were gamma adjusted to increase filopodia visualization). (JPG) [file pone.0200487.s003.jpg]

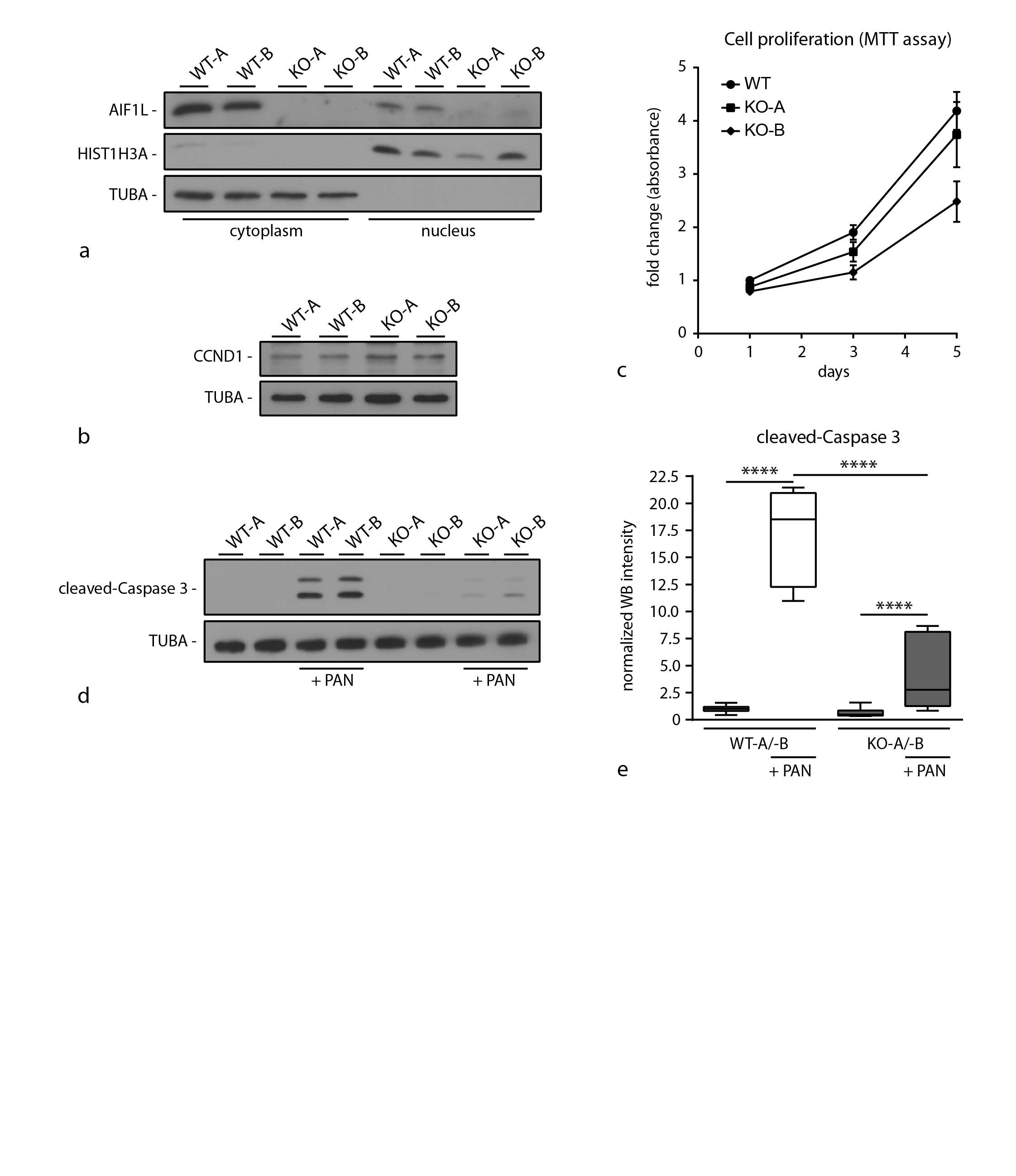

Supplement: S3 Fig — (a) Subcellular fractionation showed aside from cytoplasmic presence of AIF1L, also distinct accumulation in the nuclear compartment. The cytoplasm marker TUBA and the nuclear marker HIST1H3A demonstrate successful fractionation of these compartments. (b) Western blot for protein levels of cyclin-D1 revealed no major differences between wild type and respective AIF1L knockout clones. (c) Cell proliferation was assessed employing the MTT assay; here uniform differences in terms of proliferation were not detected (n = 3 independent experiments). (d-e) Evaluation of levels of cleaved-Caspase 3 demonstrated that knockout clones showed a lower level of accumulation of cleaved-Caspase 3 upon treatment of podocytes with the podocyte toxic agent puromycin aminonucleoside (PAN) (n = 6 WT and KO western blot (WB) intensities out of 3 independent experiments; **** p<0.0001). (JPG) [file pone.0200487.s004.jpg]

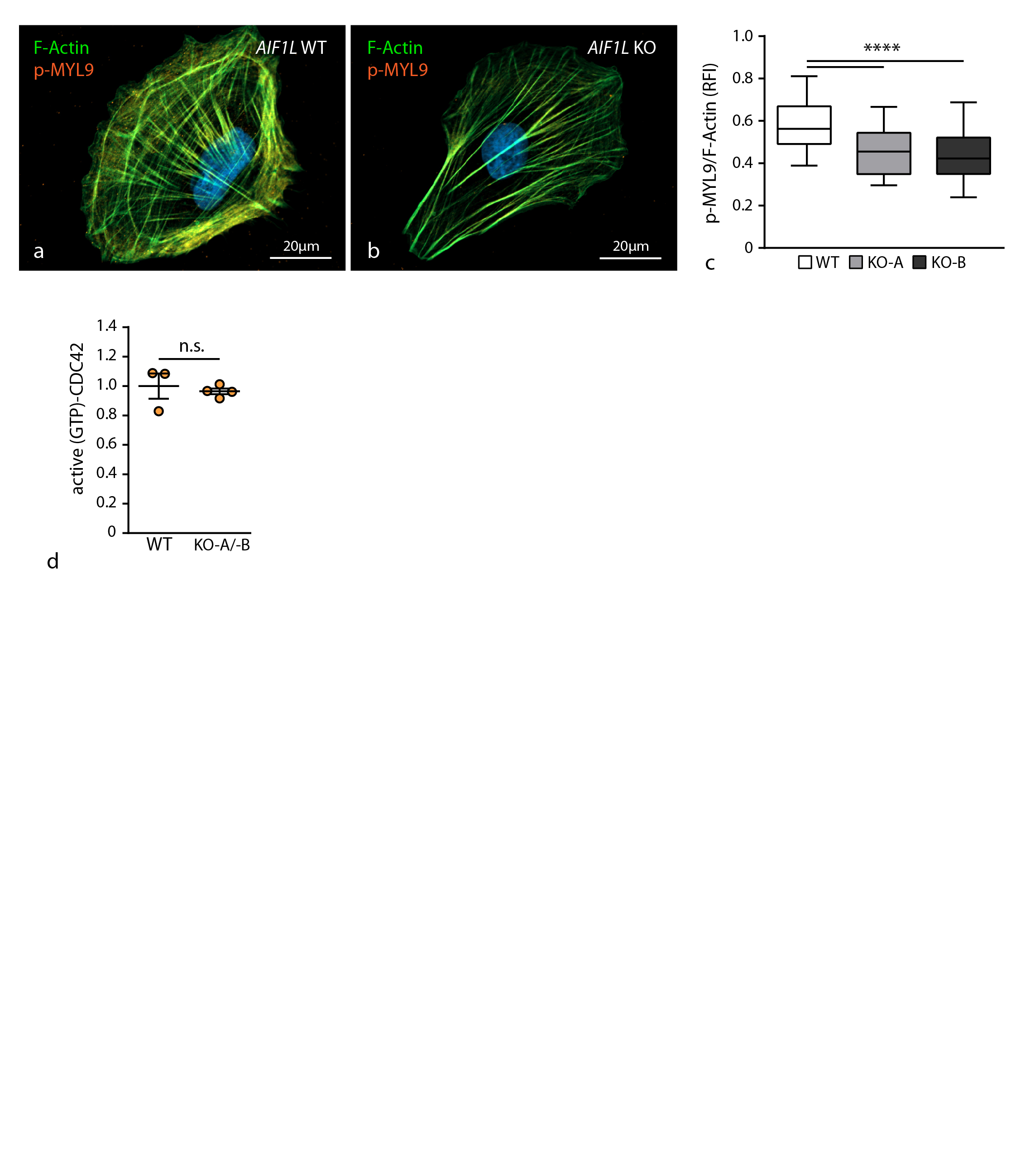

Supplement: S4 Fig — (a-c) Immunofluorescence evaluation of levels for pp-MYL9 demonstrated lower levels in respective AIF1L knockout clones when compared to wild type control cells (n = 110 WT, 61 KO-A and 64 KO-B podocytes were analyzed; **** p<0.0001). (d) Analysis of CDC42 activity by ELISA measurements could not detect any significant differences between levels of GTP-CDC42 between wild type and knockout cells (3 WT, 2 KO-A and 2 KO-B samples were analyzed; n.s—non significant). (JPG) [file pone.0200487.s005.jpg]
